# Piezo1-driven mechanotransduction as a key regulator of cartilage degradation in early osteoarthritis

**DOI:** 10.17305/bb.2024.11156

**Published:** 2024-10-07

**Authors:** Xu Yan, Su Fu, Ying Xie, Chunlin Zhang, Xuejian Wu

**Affiliations:** 1 Department of Orthopaedics, The First Affiliated Hospital of Zhengzhou University, Zhengzhou, China; 2 Department of Blood Transfusion, The First Affiliated Hospital of Zhengzhou University, Zhengzhou, China

**Keywords:** Osteoarthritis, cartilage degradation, Piezo1, mechanotransduction, catabolic activity

## Abstract

Osteoarthritis (OA) is a prevalent degenerative disease characterized by pain and cartilage damage in its later stages, while early OA is marked by the loss of cartilage’s mechanical function. Recent studies suggest that Piezo1, a mechanotransducer, may contribute to cartilage degradation under abnormal physical stress. This study investigates the mechanism by which Piezo1 mediates the loss of cartilage’s mechanical properties. Using rat chondrocytes cultured in a 3D *in vitro* model, we found that fluid flow-induced physical stress activates constitutively expressed Piezo1, leading to increased catabolic activity and apoptosis, which, in turn, disrupts the matrix structure. *Ex vivo* cartilage experiments further demonstrated that the mechanical stress-induced loss of cartilage’s physical properties (approximately 10% reduction in relaxation modulus) is mediated by Piezo1 and depends on cell viability. Notably, Piezo1 agonists alone did not alter the mechanical behavior of cartilage tissue. *In vivo*, using an OA rat model induced by anterior cruciate ligament transection, we observed cartilage integrity degradation and loss of mechanical properties, which were partially mitigated by Piezo1 inhibition. RNA sequencing revealed significant modulation of the PI3K signaling and matrix regulation pathways. Collectively, this study demonstrates that Piezo1-mediated catabolic activity in chondrocytes is a key driver of the loss of cartilage’s mechanical function during the relaxation phase.

## Introduction

Osteoarthritis (OA) is a chronic degenerative disease affecting over 300 million people globally, with its prevalence expected to rise due to aging and obesity [1]. Treatment options for OA are limited, and there is currently no cure aside from knee or hip replacement surgery. Although numerous promising therapeutic targets have been investigated, their mechanisms remain poorly understood. OA is characterized by cartilage destruction, leading to joint pain and dysfunction, typically evident in the later stages of the disease [2]. However, the loss of cartilage’s mechanical function, as indicated by changes in modulus, is an early sign of OA pathology. Mechanical properties, including elastic modulus, permeability, and relaxation strength, are typically assessed during the creep and relaxation phases of cartilage [3]. Early detection methods have shown promise [4, 5], but the molecular mechanisms underlying the loss of mechanical properties in early-stage OA remain poorly understood. Unraveling these mechanisms could reveal potential targets for early OA treatment. The etiology of OA involves factors, such as trauma, aging, obesity, and catabolic activity, with abnormal physical stress playing a key role in OA pathogenesis [2]. Healthy cartilage disperses mechanical stress and reduces friction through a hydrated matrix of aggrecan and type II collagen. In contrast, OA cartilage exhibits decreased proteoglycan content and disrupted collagen structure, leading to matrix degradation [6]. This degradation is particularly evident in cartilage from weight-bearing joints, where abnormal mechanical stimuli contribute to OA progression [7]. Chondrocytes, the sole cell type in cartilage, regulate both anabolic and catabolic processes. Changes in mechanical properties, such as stiffness and permeability, reflect cartilage degradation.

Piezo channels, discovered in 2010 [8], include Piezo1 and Piezo2, which function as mechanosensitive ion channels involved in various physiological processes [9]. Piezo1, in particular, transduces mechanical signals into cellular responses, such as OA in bone marrow stem cells [10]. In chondrocytes, Piezo1 activation by abnormal stress has been linked to senescence, apoptosis, and catabolic activity, suggesting its critical role in OA development [10–13]. Abnormal mechanical stimulation, such as compression and shear stress, leads to decreased chondrocyte activity and matrix degradation, which are closely related to OA damage. In OA cartilage tissue, Piezo1 expression is upregulated. Based on these observations, several studies have suggested that Piezo1 represents a promising therapeutic target to limit OA progression. However, whether Piezo1 mediates changes in the mechanical properties of cartilage during early OA remains unclear. By elucidating how Piezo1 influences cartilage’s mechanical behavior, particularly during the early stages of OA, this research aims to uncover potential therapeutic targets for early diagnosis and treatment. The goal is to establish the role of Piezo1 in regulating mechanical properties and catabolic activities in chondrocytes, thereby providing insights into the early detection and management of OA.

## Materials and methods

### Preparation of rat chondrocytes and cartilage explants

Healthy and clean 200 g Sprague-Dawley (SD) rats, without restriction on sex, were purchased from the Animal Center of Zhengzhou University (License number: SCXK 2021-0009). All experiments involving animals were conducted in accordance with the ethical policies and procedures approved by the Ethics Committee of the First Affiliated Hospital of Zhengzhou University. For cartilage harvest, all animals were euthanized via carbon dioxide asphyxiation. The proximal ends of the tibiae were harvested, and the cartilage tissue was cut and rapidly transferred at room temperature. The tissue was then cultured and preserved at 37 ^∘^C. Cartilage explants were placed in a 6-well plate containing 2 mL of culture medium (10% fetal bovine serum + DMEM). The cartilage tissue was processed into slices of uniform circular area, described as cartilage explants, which were prepared for *ex vivo* experiments and mechanical testing. For chondrocyte isolation, the cartilage tissue was collected and digested with enzymes. The cartilage was diced before being transferred to a conical tube and incubated for 1 h in a 37 ^∘^C oven on a roller, with 7 units/mL pronase prepared in culture media. After pronase was carefully aspirated, the tissue was incubated in 100 units/mL collagenase in the same conditions for an additional 16 h. After discarding the supernatant, the cell pellet was re-suspended in culture media with 10% fetal bovine serum. Pre-autoclaved agarose gel was placed in an oven at 80 ^∘^C for 20 min and then allowed to cool naturally. The chondrocyte suspension was mixed with an equal volume of molten 6% (w/v) agarose, creating constructs with cells in 3% (w/v) agarose, molded for further experiments. Constructs were prepared for staining, while for RNA/protein detection and apoptosis assays, the specimens were digested using papainase solution.

### Apoptosis assay

Apoptosis of induced cells was assessed using Annexin V-FITC labeling and analyzed by flow cytometry. The ratio of apoptotic cells was calculated by dividing the number of positive cells by the total number of cells. The working solution of Annexin V-FITC was prepared immediately before use. Treated cells were washed three times with PBS and resuspended in binding buffer. Flow cytometric analysis was performed immediately following incubation with 100 ng/mL Annexin V-FITC in the dark for 10 min. The cells were then incubated with 5 mg/mL of a secondary stain for 5 min, washed three times with PBS, and examined.

### Animals and experimental design

OA was induced in SD rats via anterior cruciate ligament (ACL) transection, a widely applied method [1]. The rat knee joint was exposed under inhalational anesthesia, followed by the resection of the ACL. The contralateral capsule was cut as a sham control. Rats were randomly assigned to the Sham group, OA model group, or OA model + intra-articular injection of GsMTx4 group. In the Sham group, the procedure only included a skin incision to expose the cartilage, and 100 µL of saline was injected into the articular space weekly. In the OA model + GsMTx4 group, 100 µL of 40 µM GsMTx4 was injected intra-articularly weekly [14]. Six weeks after surgery, the right knee joints of the rats were collected. After fixation in 4% paraformaldehyde and decalcification with 0.5 M EDTA, the tissue was sliced for staining. Meanwhile, total RNA was collected using a lysis buffer and subjected to RNA sequencing.

### Mechanical loading of shear stress by fluid flow

The method for generating shear stress by fluid flow in cartilage explants or cells has been previously reported [15]. The cells/agarose constructs and cartilage tissue were 0.25 mm in radius and 1 mm in thickness. A fluid flow stress device, created using a 3D printer, produced several small chambers. After sterilization, agarose in 2% weight/volume PBS was placed until fully gelled. The mold was then removed, and the constructs were placed into the small chambers. The device, filled with culture media, was placed on an orbital shaker in an incubator. The exact fluid flow stress was calculated using ANSYS software, with the rotator set at 100 RPM to induce fluid flow and produce shear stress for 48 h.

### Detection of physical properties of cartilage tissue

The mechanical properties or behavior of cartilage tissue have been widely explored using various devices, such as strain detectors, to compress cartilage explants. Due to the small size of rat cartilage, we employed a microindentation test, as previously reported [16]. Briefly, the proximal part of the rat tibia was removed and embedded in a fixing ring with adhesive gel. A drop of culture medium was applied to the top of the cartilage tissue to maintain its water content. A set of testing programs and appropriate probes from the indentation test system were prepared. During indentation, the displacement of the indenter was recorded at a cross-read speed necessary to generate the regulated strain and strain rate. Each explant was pre-loaded to 0.01 N to ensure direct contact, with displacement documented. The cartilage tissue was then subjected to a 20% compressive strain applied at a strain rate of 20%/min (creep phase). For example, a cartilage slice with a thickness of 200 µm was compressed by 40 µm within 1 min. A recovery period of 4 min was allowed by maintaining the compressor in the same location. Strain values were recorded every second to generate a scatter plot for further analysis.

### Live/dead staining of cartilage tissue

The viability of chondrocytes was assessed using a live/dead staining kit consisting of Calcein AM (Sigma) and Ethidium homodimer-1 (EthD-1, Fisher Scientific). The tissue was immersed in a medium containing 5 µM Calcein AM and 5 µM EthD-1 for 30 min at 37 ^∘^C. After washing with PBS three times, the tissue was placed on coverslips and immediately imaged using a microscope with a 10× objective. Calcein AM labeled live chondrocytes green (excitation 495 nm, emission 515 nm), while EthD-1 labeled dead cells red (excitation 528 nm, emission 617 nm).

### RNA-sequencing analysis of cartilage tissue

Cartilage tissue was harvested, and total mRNA was isolated using TRIzol reagent (Invitrogen) according to the manufacturer’s protocol. The enriched mRNAs were reverse transcribed into cDNA using random primers. Second-strand cDNA was synthesized using DNA polymerase I, RNase H, dNTPs, and buffer. The cDNA fragments were purified using the QiaQuick PCR extraction kit, end-repaired, and ligated to Illumina sequencing adapters. The digested products were size-selected by agarose gel electrophoresis, PCR-amplified, and sequenced. Six cDNA libraries were constructed using the Illumina NovaSeq 6000 system by Gene Denovo Biotechnology Co., (Guangzhou, China). Results from gene ontology (GO) analysis were subsequently analyzed and utilized.

### Histological staining

Serial sagittal sections of rat samples were cut at 6 µm and stained with H&E, Alcian Blue, and Safranin-O. Based on the images, Osteoarthritis Research Society International (OARSI) scoring was used to assess the severity of OA. For immunohistochemistry, sections were incubated overnight at 4 ^∘^C with anti-Piezo1 (1:1000). Sections were visualized using an HRP system, and the percentage of positive cells was quantified by two blinded pathologists.

### Quantitative polymerase chain reaction (q-PCR)

To validate osteogenic gene expression, total RNA was isolated from chondrocytes or cartilage tissue using an isolation column from Qiagen (74004). cDNA was synthesized using TRIzol and Oligo-dT (15596026, SO132 from Invitrogen). In some cases, the concentration and quality of RNA differed significantly between samples. PCR was performed with the SYBR Green assay kit (A25779 from Invitrogen), following the amplification protocol: 95 ^∘^C for 3 min, followed by 40 cycles alternating between 95 ^∘^C for 15 s and 60 ^∘^C for 30 s. Primers were designed based on sequences in GenBank (primer sequences listed below: MMP-13: F-CTTGATGCCATTACCAGTC, R-GGTTGGGAAGTTCTGGCCAADAMTs5: F-TATGACAAGTGCGGAGTATG, R-TTCAGGGCTAAATAGGCAGTCollagen: F-ACGTCCAGATGACCTTCCTG, R-GGATGAGCAGAGCCTTCTTGAgg: F-GAGTTTGTCAACAACAATGCC, R-TGGTAATTACATGGGACATCGGAPDH: F-GACAAAATGGTGAAGGTCGG, R-TCCACGACATACTCAGCACC; all 100–300 bp in length).The fold changes in gene expression were analyzed using the 2^-ΔΔCT^ method and normalized to GAPDH expression. The reported data represent the mean expression from three experiments.

### Western blot analysis

Western blotting was used to determine the protein expression levels of Piezo1, matrix metalloproteinase-13 (MMP-13), thrombospondin motifs 5 (ADAMTs5), Aggrecan, and Collagen II. Cells or tissue samples were extracted and dissolved in 150 µL of lysis buffer supplemented with 1% (v/v) protease inhibitor cocktail (78440 from Invitrogen). Proteins were separated using SDS-PAGE and transferred to membranes (1704156 and 1620176 from Bio-Rad). After blocking (37565 from Invitrogen), membranes were stained with primary antibodies against tubulin, Piezo1, MMP-13, ADAMTs5, Aggrecan, and Collagen II, all purchased from Santa Cruz Biotechnology. Membranes were then incubated overnight with horseradish peroxidase-conjugated secondary antibodies (31460 and 31430 from Invitrogen) and visualized by chemiluminescence. ImageJ was used to quantify the intensity of target protein bands in each blot.

### Ethical statement

This study was reviewed and approved by the Ethics Committee of the First Affiliated Hospital of Zhengzhou University. No patients were involved in this study.

### Statistical analysis

GraphPad Prism was used to conduct the statistical analyses. Gene/protein expression data and mechanical test data are presented as the means ± SD from at least three repeats and were analyzed using Student’s *t*-tests for two separate groups. For comparisons involving more than two groups, one-way ANOVA followed by Tukey’s post hoc tests was used. Fisher’s exact test was applied for frequency data. The significance level was set to *P* < 0.05.

**Figure 1.**
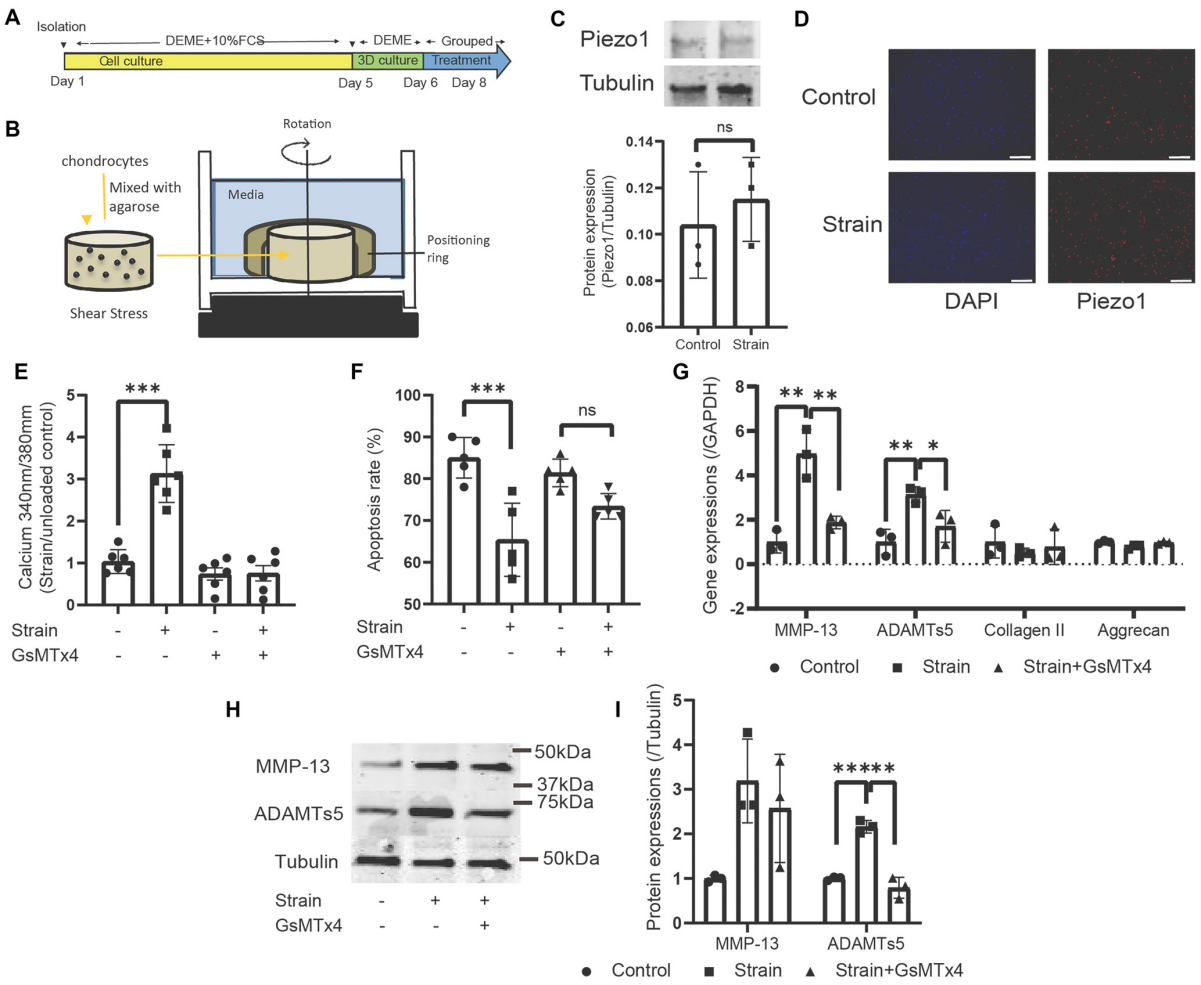
**Mechanical strain activated Piezo1-Ca^2+^ influx to induce apoptosis and catabolic activity in chondrocytes cultured in a 3D construct *in vitro*.** Isolated chondrocytes cultured in a 3D construct were unloaded, loaded, or loaded with GxMTx4 after culture and serum deprivation, as illustrated in (A). The mechanical loading pattern using a specialized device is shown in (B). Piezo1 protein expression in unloaded and loaded conditions is displayed in (C). Additionally, immunofluorescence labeling of Piezo1 in cell constructs is presented for both unloaded and loaded cells (D). (E) Ca^2+^ influx in response to strain in cells without and with GxMTx4. The apoptosis frequency of cells under unloaded or loaded conditions, with and without GxMTx4, is shown in (F). mRNA isolated from cells was analyzed by qRT-PCR (G), showing the gene expression levels of MMP-13, ADAMTs5, Aggrecan, and Collagen II, normalized to the unloaded control. The protein bands and expressions of MMP-13, ADAMTs5, and Tubulin are shown in (H) and (I). Scattered dots with bars represent the means ± SD (*N* ═ 3 in (C), *N* ═ 6 in (E), *N* ═ 5 in (G), and *N* ═ 3 in (H) and (I)). Statistically significant differences were determined using Student’s *t*-tests for (C) and one-way ANOVA for ((E)–(G) and (J)). *P < 0.05, **P < 0.01, ***P < 0.001 indicate statistically significant differences. The symbol “ns” indicates no significant difference. MMP-13: Matrix metalloproteinase-13.

## Results

### Shear stress stimulated catabolic response and apoptosis via Piezo1 mediating calcium influx in 3D cultured chondrocytes *in vitro*

*In vitro* experiments identified the harmful effects of mechanical stress induced by fluid flow on chondrocyte metabolism and apoptosis. Figure 1A shows the experimental protocol where rat chondrocytes were harvested by enzyme digestion on Day 1, followed by a 5-day monolayer culture. The cells were then reseeded in a 3D construct with 3% w/v agarose gel. They were either placed in static control conditions or subjected to shear stress from fluid flow for 48 h. The mechanism of shear stress conduction is depicted in Figure 1B, simulating shear strain using a 100 RPM orbital shaker, which was also applied to the cartilage explants. In both unloaded and loaded cells, Piezo1 was stably expressed, with no changes detected by western blot analysis (Figure 1C). Immunofluorescence and DAPI staining showed Piezo1 localization in the nuclear area (Figure 1D). In response to shear stress, Ca^2+^ influx was monitored using the Fura-2 AM indicator. Figure 1E demonstrates the calcium influx initiated by mechanical loading, while Piezo1 inhibitor GsMTx4 blocked this effect, indicating that Piezo1 is required for mechanical calcium signaling. Apoptotic cells were assessed by flow cytometry, revealing that mechanical loading induced apoptosis, which was blocked by Piezo1 inhibition (Figure 1F). Interestingly, pathological stress activated Piezo1, elevating catabolic activities at the gene transcription and protein expression levels. Mechanically regulated catabolic genes, such as MMP-13 and ADAMTs5, showed increased expression, which was reduced after Piezo1 inhibition (Figure 1G). Specifically, 4.9 ± 1.1 and 3.1 ± 0.4-fold changes were observed for MMP-13 and ADAMTs5, respectively, following mechanical loading, but these were reduced to 1.9 ± 0.3 and 1.7 ± 0.7-fold changes with Piezo1 inhibition. The changes in MMP-13 and ADAMTs5 protein levels mirrored the gene expression results, further indicating that Piezo1 mediates mechanically induced catabolic activity in chondrocytes (Figure 1H). A 2.2 ± 0.2-fold increase in ADAMTs5 protein expression was observed in loaded cells, while Piezo1 antagonism significantly reduced ADAMTs5 expression to control levels (Figure 1I).

**Figure 2.**
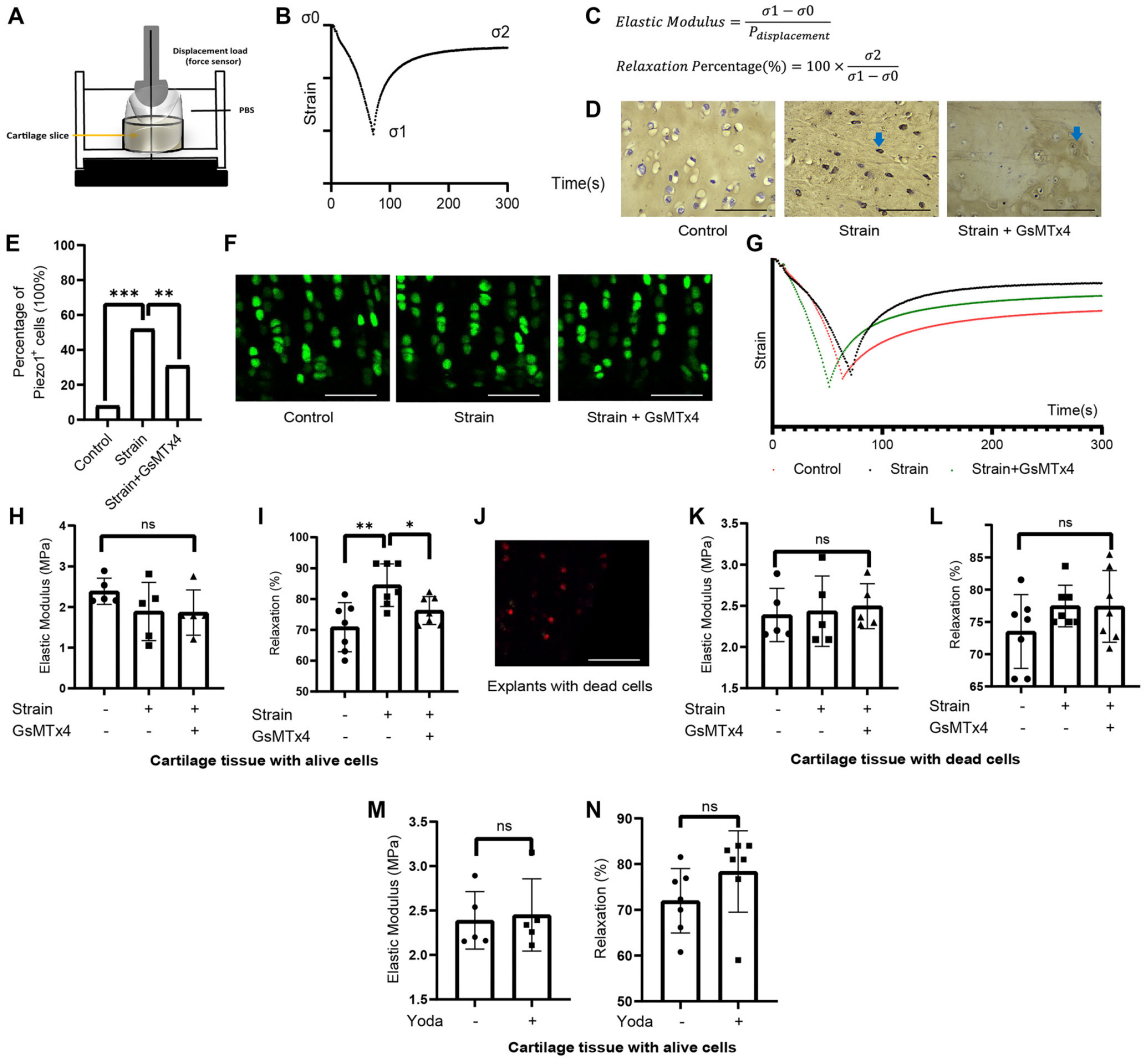
**Loss of mechanical properties during the relaxation phase due to mechanical loading in cartilage explants *ex vivo* was mediated by Piezo1-regulated live chondrocytes.** The method for mechanical testing is shown in (A), where compression strain over time was analyzed during the creep and relaxation phases (B). Based on the equations (C), the modulus during both the elastic and relaxation phases was calculated, along with the percentage of relaxation. Cartilage explants were either unloaded, loaded with shear stress, or strain-loaded with additional GsMTx4. Piezo1 expression within the tissue was first detected (D), with the frequency of positive cells shown (E). Meanwhile, live cells within the tissue were identified (F). The representative curve during mechanical testing is shown in (G). For explants with live cells, the elastic modulus (H) and percentage of relaxation (I) were calculated, showing significant differences. In cartilage explants with all dead cells, confirmed by live/dead staining (J), the mechanical behavior for elastic modulus (K) and percentage of relaxation (L) is also shown. In explants with and without Yoda1 treatment, mechanical properties remained unchanged for modulus (M) and relaxation percentage (N). Scattered dots with bars represent the means ± SD (*N* ═ 5 in (H), (K), and (M), *N* ═ 7 in (I), (L), and (N)). Statistically significant differences were determined using Mann–Whitney tests ((H), (K), (M), (I), (L), and (N)) and Fisher’s exact test (E). *P < 0.05, **P < 0.01, ***P < 0.001 indicate significant differences. “ns” indicates no significant difference.

### Piezo1 mediated the loss of mechanical properties during relaxation phase in cartilage explants *ex vivo* via regulating live chondrocytes

Cartilage tissue was harvested from rat knees and cut into explants for mechanical testing *ex vivo*. The device used for mechanical testing is shown in Figure 2A. The compression strain over time was conducted through the creep and relaxation phases. Figure 2B illustrates the creep behavior from σ0 to σ1 (peak strain) and the relaxation phase from σ1 to σ2 (final stable condition). Based on the equations (Figure 2C), the elastic modulus and relaxation percentage of the cartilage tissue were measured. Before mechanical testing, Piezo1 expression within the tissue was detected. Piezo1-positive cells were rarely seen in the control, while Piezo1-positive cells were frequently observed in the strained tissue (blue arrow, Figure 2D). This difference was statistically significant, as shown in Figure 2E. Live/dead staining (Figure 2F) revealed that cell viability was not affected by the treatment, indicating that apoptosis was differently influenced in 3D cultures compared to cartilage tissue. The representative strain–time plots for explants from three groups are shown in the scatter plot (Figure 2G). The elastic modulus and relaxation modulus were maintained, but the relaxation percentage differed significantly. In the unloaded, loaded, and GsMTx4-treated loaded cartilage explants, the elastic modulus was approximately 2 MPa with no significant differences between the groups (Figure 2H). Notably, the relaxation percentage increased from 70.9 ± 8.0% in the control to 84.5 ± 6.7% in the loaded tissue, but was restored to 77.1 ± 4.4% with additional GsMTx4 treatment (Figure 2I). This alteration in mechanical properties reflects the matrix integrity of the cartilage. To test the role of chondrocytes, mechanical tests were performed on cartilage tissue without live cells (as shown in Figure 2J). Mechanical properties from both the creep and relaxation phases remained unchanged (Figure 2K and 2I), demonstrating that live chondrocytes are required for the mechanically regulated loss of physical properties under injury. It was proposed that the metabolic activity of live cells plays a significant role in the Piezo1 mechanism. In contrast, Piezo1 activation with Yoda1 alone did not alter mechanical behavior in terms of elastic modulus (Figure 2M) or relaxation percentage (Figure 2N), suggesting that Piezo1 plays a regulatory role, but not an initial role, in the loss of mechanical properties.

### Piezo1 mediated the physical properties loss of cartilage in rat OA model *in vivo*

**Figure 3.**
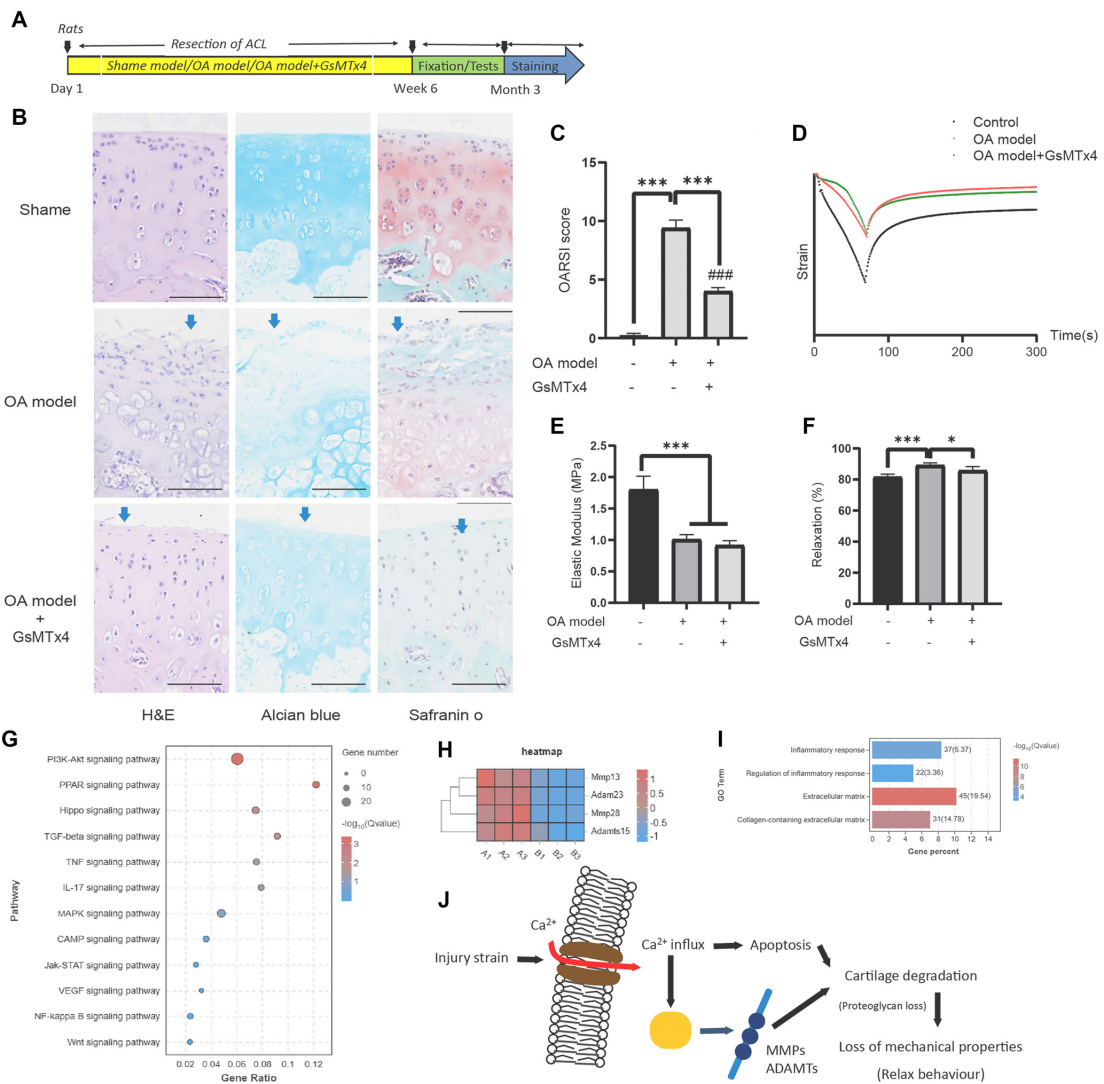
**Piezo1 mediated the loss of cartilage physical properties in a rat OA model *in vivo*.** The OA model was created and divided into three groups: sham control, OA model, and OA model with intra-articular injection of GsMTx4 (A). The results from H&E, Alcian Blue, and Safranin-O stains (B) demonstrated the OA lesions in the three groups. The OARSI scores from each staining also reflected this trend (C). As shown in the representative strain curve (D), the elastic modulus (E) and relaxation percentage (F) indicated the protective role of the Piezo1 antagonist against the mechanical loss of cartilage. RNA-sequencing data indicated several possible pathways (G), differentially expressed genes (H), and part of the GO analysis results on matrix regulation (I). The final mechanism of Piezo1’s effect on mechanical properties is illustrated in the schematic drawing (J). Bars represent the means ± SD (*N* ═ 3 in (C), *N* ═ 6 in (F) and (G)). Statistically significant differences were determined using one-way ANOVA ((C), (F), and (G)). *P < 0.05, ***P < 0.001 indicate significant differences. The symbol “ns” denotes no statistical difference. OA: Osteoarthritis; GO: Gene ontology; OARSI: Osteoarthritis Research Society International.

We further investigated the effects of a Piezo1 antagonist on OA damage in rats. The OA model was induced on Day 1 by resection of the ACL in the rat knee, and the experiment continued for the following six weeks (Figure 3A). Rats were divided into three groups: sham control, OA model, and OA model with intra-articular injection of GsMTx4. At the end of the study, cartilage tissue was either fixed for staining or collected for RNA-sequencing analysis. Results from H&E, Alcian Blue, and Safranin-O staining (Figure 3B) showed that the OA model markedly exacerbated lesions in the knee joints, while GsMTx4 injection exhibited a protective effect. Specifically, the collagen–proteoglycan structure was disrupted (blue arrows in the OA model group, Figure 3B), and collagen content was decreased. Additional treatment with GsMTx4 mitigated OA lesions, though small, localized cartilage injuries were still observed (blue arrows, Figure 3B). OARSI scores from the three types of staining reflected this trend, showing that the score in control rats was lower than in the OA model group and the OA model + GsMTx4 group (Figure 3C). A significant difference between the latter two groups indicated that Piezo1 modulation partially protected the cartilage in the OA model. In terms of the mechanical tests, representative strain–time plots for cartilage tissue from the three groups are shown in the scatter plot (Figure 3D). The elastic modulus of the cartilage was significantly decreased in the OA model, and Piezo1 inhibition did not reverse this effect (Figure 3E). Interestingly, the relaxation behavior of the cartilage differed between groups. The relaxation percentage (81.7 ± 1.8% in control) was up-regulated to 89.1 ± 1.6% in the OA model, but was reduced to 85.7 ± 2.6% after GsMTx4 treatment (Figure 3F). RNA-sequencing data provided additional insights, suggesting potential involvement of pathways, such as PI3K signaling, PPAR signaling, and Hippo signaling pathways, according to KEGG analysis (Figure 3G). These signaling pathways may contribute to Piezo1-mediated loss of cartilage mechanical properties during the relaxation phase by regulating the “Piezo1 – matrix degradation – cartilage mechanical behavior” axis. Differentially expressed genes related to bone metabolism, including MMPs and ADAMTs, were involved (Figure 3H and 3I), with GO analysis highlighting the enrichment of genes involved in matrix regulation.

## Discussion

This study highlights the crucial role of the mechanosensitive Piezo1 channel in the loss of cartilage’s physical properties. Our findings from *in vitro*, *ex vivo*, and *in vivo* experiments demonstrate that Piezo1 activation by shear stress enhances the catabolic activity of chondrocytes, thereby affecting the relaxation behavior of cartilage during mechanical testing. At the molecular level, shear stress induces apoptosis and upregulates catabolic genes, such as MMP-13 and ADAMTS5 in chondrocytes through Piezo1-mediated Ca^2+^ influx. *Ex vivo*, shear stress increases the proportion of Piezo1(+) cells while maintaining cell viability. Although shear stress impairs the relaxation modulus, this effect is counteracted by Piezo1 inhibition with GsMTx4, while no significant impact on the elastic modulus was observed. *In vivo*, GsMTx4 injection prevents OA progression but only restores the relaxation behavior of cartilage. RNA sequencing reveals that PI3K signaling and matrix regulation pathways involving MMPs and ADAMTSs modulate inflammatory responses and matrix stability. Thus, Piezo1-mediated loss of mechanical relaxation properties in cartilage is evident. Mechanical loading through shear stress can have both beneficial and detrimental effects on cartilage homeostasis. While low-frequency shear stress promotes proteoglycan and collagen synthesis, excessive shear stress is harmful [17]. For instance, shear stress exceeding 0.21 Pa has been shown to damage cartilage [15]. In this study, we focused on injury-level shear stress to explore the Piezo1-mediated catabolic effects on cartilage. The shear stress was approximately 1–2 Pa, as estimated by ANSYS software. We designed and applied a loading machine to simulate abnormal mechanical loading, which is characteristic of OA pathology [18]. Although other forms of mechanical loading, such as tensile strain and compression, could be applied, shear stress was consistently used to load 3D chondrocyte constructs and explants throughout the study due to practical constraints. Piezo1 is widely recognized for its mechanical responsiveness and its role in regulating OA lesions and osteophyte formation [12–14]. Previous studies have shown that knocking out the Piezo1 gene or administering GsMTx4 can prevent OA progression [14], which aligns with our findings. However, our study further delved into the mechanical behavior of cartilage tissue. Cartilage’s viscoelastic properties, characterized by creep and relaxation phases, are early indicators of OA pathology [4, 5]. Although we did not find evidence that Piezo1 regulates creep behavior, this might be due to the compressive modulus formed by negatively charged proteoglycans, which Piezo1 minimally affects. This aligns with the observed loss of supportive force in OA cartilage during joint movement. Previous studies have reported that the elastic modulus of cartilage varies depending on loading conditions, with rat cartilage showing an elastic modulus of approximately 2 MPa, consistent with studies using similar methodologies [16]. In contrast, relaxation responses, which are tied to matrix integrity—specifically the collagen–proteoglycan matrix—are crucial for preventing crack nucleation in cartilage [19]. Previous studies have demonstrated that Piezo1 inhibition by siRNA reduces the expression of MMP-13 and ADAMTS5, both key regulators of cartilage degradation. We demonstrated that Piezo1 governs the relaxation response through factors like MMP-13 and ADAMTS5, while RNA-sequencing data suggested additional pathways, such as PI3K and Col6. Piezo channels are well-characterized in terms of their structure and function [8, 9], participating in various biological and pathological processes, including vascular mechanobiology [20], erythrocyte volume regulation, and sensing pressure changes in the genitourinary tract [21]. Recent studies have highlighted the role of Piezo1 in bone homeostasis, showing its necessity for bone formation by mediating osteogenesis in osteoblasts [22] and facilitating osteoblast–osteoclast crosstalk [23]. However, unlike its anabolic role in bone, Piezo1 seems to mediate OA progression in chondrocytes, likely due to differences in cell types. Mechanical loading triggers intracellular calcium influx via Piezo1, activating the calcineurin/NFAT signaling pathway [24], which mediates OA lesions. Other ion channels may also be involved; for example, strain-induced calcium influx through transient receptor potential vanilloid 4 (TRPV4) is another major contributor [25, 26]. Additionally, harmful mechanical strain predominantly induces calcium influx through the Piezo2 channel. YAP, a known mechanotransducer, may act as a downstream factor [27]. Previous studies have linked Piezo1-mediated mechanical responses to the Wnt/β-catenin signaling pathway, with elevated Wnt/Ctnnb1 expression identified through KEGG pathway mapping [24]. Our RNA-sequencing results are consistent with these observations. The findings of this study also have significant implications for cell-based therapies in the treatment of OA. Given that Piezo1 activation contributes to cartilage degradation by transducing mechanical injury signals and compromising the extracellular matrix, targeting Piezo1 could enhance the efficacy of cell-based approaches. For instance, mesenchymal stem cells (MSCs) or chondrocyte-based therapies could potentially be optimized by either modulating Piezo1 activity or engineering cells with reduced Piezo1 expression. This could protect newly formed cartilage from mechanical stress-induced degradation, thereby improving tissue integration and longevity. This study has several limitations. First, more molecular pathways involved in Piezo1-regulated events need to be elucidated, as some factors may directly affect the mechanical behavior of cartilage. Second, we used shear stress to monitor cell responses to mechanical loading. Other specific forms of mechanical loading, such as compression, tensile strain, and hypostatic pressure, both dynamic and static, could lead to varying responses. Lastly, the clinical implications of Piezo1-mediated mechanical behavior in OA patients remain to be explored in future research.

## Conclusion

In conclusion, this study highlights the role of Piezo1 activation in the deterioration of cartilage mechanical properties during OA pathogenesis. Piezo1 transduces mechanical injury signals, leading to extracellular matrix degradation and a reduction in cartilage’s relaxation capacity. These findings enhance our understanding of the early stages of OA and hold important implications for the development of drugs and treatments aimed at modulating OA progression.

## Data Availability

The datasets used and/or analyzed during the current study are available from the corresponding author on reasonable request.
